# Anxiety among healthcare workers infected by COVID-19: A cross-sectional study

**DOI:** 10.1192/j.eurpsy.2026.11025

**Published:** 2026-07-31

**Authors:** A. Aloui, N. Ganoun, A. chouchane, I. Kacem, N. Zommit, M. Bouhoula, M. Makhloufi, H. Kalboussi, O. El Maalel, S. Chatti

**Affiliations:** 1 Department of Occupational Medicine; 2 Departement of Epidemiology, Farhat Hached university hospital, Sousse, Tunisia

## Abstract

**Introduction:**

The impact of COVID-19 on mental health has been continuously reported, especially among healthcare workers. In the literature, anxiety symptoms are not uncommon in infected individuals. However, there is a little data on these psychological events in healthcare professionals.

**Objectives:**

To determine the prevalence and the associated factors of anxiety symptoms among healthcare workers infected by COVID-19.

**Methods:**

This is a cross-sectional study conducted among healthcare workers at Farhat Hached University Hospital infected by COVID 19. A self-administered questionnaire was used to collect data from the socio-professional and medical characteristics of the participants. Anxiety symptoms were assessed by the Hospital Anxiety and Depression Scale (HAD-S).

**Results:**

A total of 477 confirmed COVID-19 in healthcare works were included in this study (85.9%). The mean age of the participants was 39.9 ±10.8 years. Women represented 78.2%. The majority of the infected participants were nurses (32.1%). The pauci-symptomatic form of the disease was the most frequent (73.8%). After returning to work, 62.7% of the participants retained residual symptoms and 15.5% experienced stigma reaction from their colleagues. The overall prevalence of anxiety symptoms were 25.4%. Furthermore, residual symptoms and duration of confinement predicted anxiety symptomatology.

**Conclusions:**

The psychological events of COVID-19 are frequent among healthcare workers. Thus systematic screening and early management of psychological disorders are necessary to preserve the human resources of the health sector.

**Disclosure of Interest:**

None Declared

